# Occurrence of virulent multidrug-resistant *Enterococcus faecalis* and *Enterococcus faecium* in the pigs, farmers and farm environments in Malaysia

**DOI:** 10.7717/peerj.5353

**Published:** 2018-08-06

**Authors:** Shiang Chiet Tan, Chun Wie Chong, Cindy Shuan Ju Teh, Peck Toung Ooi, Kwai Lin Thong

**Affiliations:** 1 Institute of Biological Science, Faculty of Science, University of Malaya, Kuala Lumpur, Malaysia; 2 Department of Life Sciences, School of Pharmacy, International Medical University, Kuala Lumpur, Malaysia; 3 Centre for Translational Research, Institute for Research, Development and Innovation, International Medical University, Kuala Lumpur, Malaysia; 4 Department of Medical Microbiology, Faculty of Medicine, University of Malaya, Kuala Lumpur, Malaysia; 5 Department of Veterinary Clinical Studies, Faculty of Veterinary Medicine, Universiti Putra Malaysia, Selangor Darul Ehsan, Malaysia

**Keywords:** *Enterococcus faecium*, *Enterococcus faecalis*, MDR, Farmers, Virulence genes, Pigs

## Abstract

**Background:**

*Enterococcus faecalis* and *Enterococcus faecium* are ubiquitous opportunistic pathogens found in the guts of humans and farmed animals. This study aimed to determine the occurrence, antimicrobial resistance, virulence, biofilm-forming ability and genotypes of *E. faecalis* and *E. faecium* from swine farms. Correlations between the genotypes, virulotypes, antibiotic resistance, and the environmental factors such as locality of farms and farm hygiene practice were explored.

**Methods:**

*E. faecalis* and *E. faecium* strains were isolated from the oral, rectal and fecal samples of 140 pigs; nasal, urine and fecal samples of 34 farmers working in the farms and 42 environmental samples collected from seven swine farms located in Peninsular Malaysia. Antibiotic susceptibility test was performed using the disk diffusion method, and the antibiotic resistance and virulence genes were detected by Polymerase Chain Reaction. Repetitive Extragenic Palindromic-Polymerase Chain Reaction and Pulsed-Field Gel Electrophoresis were performed to determine the clonality of the strains. Crosstab/Chi-square test and DistLM statistical analyses methods were used to determine the correlations between the genotypes, virulence factors, antibiotic resistance, and the environmental factors.

**Results:**

A total of 211 *E. faecalis* and 42 *E. faecium* were recovered from 140 pigs, 34 farmers and 42 environmental samples collected from seven swine farms in Peninsular Malaysia. Ninety-eight percent of the strains were multidrug-resistant (resistant to chloramphenicol, tetracycline, ciprofloxacin and erythromycin). Fifty-two percent of the strains formed biofilms. Virulence genes *efa, asa*I*, gel*E, *esp*, *cyl* and *ace* genes were detected. Virulence genes *efa* and *asa*I were most prevalent in *E. faecalis* (90%) and *E. faecium* (43%), respectively. Cluster analyses based on REP-PCR and PFGE showed the strains were genetically diverse. Overall, the strains isolated from pigs and farmers were distinct, except for three highly similar strains found in pigs and farmers. The strains were regional- and host-specific.

**Discussion:**

This study revealed alarming high frequencies of multidrug-resistant enterococci in pigs and swine farmers. The presence of resistance and virulence genes and the ability to form biofilm further enhance the persistence and pathogenicity of the strains. Although the overall clonality of the strains were regionals and host-specific, strains with high similarity were found in different hosts. This study reiterates a need of a more stringent regulation to ensure the proper use of antibiotics in swine husbandry to reduce the wide spread of multidrug-resistant strains.

## Introduction

Enterococci are Gram-positive facultative anaerobes and are part of microbiota of humans and animals (Lebreton, Willems & Gilmore, 2014; Kristich, Rice & Arias, 2014). Antibiotics such as macrolides, trimethroprim, fluoroquinolones and tetracyclines are commonly used for animal husbandry and human medicine in Southeast Asia, and resistances towards these antibiotics have been reported for *Enterococcus* spp. (Daniel et al., 2015; Health Action International Asia Pacific, 2013).

Livestock industry plays an important role in the transmission of multidrug-resistant (MDR) *Enterococci* strains due to the close interaction between farmers, livestock and the farm environment (Novais et al., 2013; Woolhouse et al., 2015). Antimicrobial agents are used as prophylactics in feeds and the pervasive selection of resistant bacteria in the swine gut facilitates the persistence and dissemination of MDR strains to other animals and humans (Woolhouse et al., 2015; Hwang et al., 2009). The emergence of MDR bacteria in the livestock industry is of public health concern as transmission of these bacteria to humans has been reported (Compassion in World Farming, 2011). The spread of such MDR strains can occur through direct (consumption of contaminated food, direct contact of farmers and veterinarians) or indirect (animal waste handling, contaminated ground water or surfaces) routes (Daniel et al., 2015). The resistance determinants carried by the MDR strains could also be transmitted to the other commensal strains in the host and cause further complications (Price et al., 2018). In addition, infections caused by MDR strains have been associated with long hospital stays, high morbidity and mortality rate (Beganovic et al., 2018).

The two most common enterococcal species, *Enterococcus faecalis* and *Enterococcus faecium*, present diverse virulence factors such as aggregation substance, gelatinase, cytolysin, enterococcal surface protein and collagen binding cell wall protein which are encoded by their respective genes *asa*1*, gel*E*, cyl*A*, esp* and *ace* (in plasmids) or *hyl* (in chromosome) (Vidana et al., 2015). Enterococcal surface protein, encoded by *esp* gene, is one of the factors associated with biofilm formation (Toledo-arana et al., 2001). The biofilm formation ability can enhance the persistence and endurance of the strains by providing extra protection to the bacteria cells and confers higher tolerance to the antibiotics (Holmberg & Rasmussen, 2016).

The objectives of the study were to determine the occurrence of MDR *E. faecalis* and *E. faecium* isolated from pigs, swine farmers and swine farm environment in Peninsular Malaysia. The virulotypes, biofilm-forming ability, and the genotypes (based on PFGE and REP-PCR) were determined.

## Materials and Methods

### Sample collection

**Figure 1:**
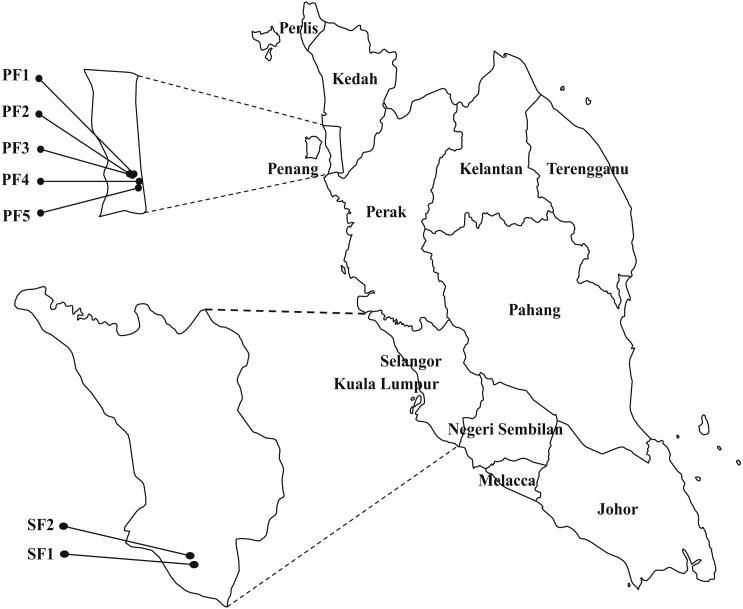
The map of Peninsular Malaysia showing the locations of the seven swine farms. Number of strains in each farm: PF1 (*n*_*E*. *faecalis*_ = 40; *n*_*E*. *faecium*_ = 3); PF2 (*n*_*E*. *faecalis*_ = 31; *n*_*E*. *faecium*_ = 7); PF3 (*n*_*E*. *faecalis*_ = 13; *n*_*E*. *faecium*_ = 0); PF4 (*n*_*E*. *faecalis*_ = 24; *n*_*E*. *faecium*_ = 4); PF5 (*n*_*E*. *faecalis*_ = 12; *n*_*E*. *faecium*_ = 1); SF1 (*n*_*E*. *faecalis*_ = 43; *n*_*E*. *faecium*_ = 23); SF2 (*n*_*E*. *faecalis*_ = 48; *n*_*E*. *faecium*_ = 4).

Samplings were carried between August to December 2013 in seven selected swine farms in the northern (PF1, PF2, PF3, PF4 and PF5) and central (SF1 and SF2) regions of Peninsular Malaysia (Fig. 1). The selected regions are high density pig farming areas. Ten piglets and 10 weaning pigs were randomly selected from each farm and 140 oral swabs, 140 rectal swabs and 91 fecal samples were collected from 140 pigs from seven swine farms. All samples were collected under veterinary supervision. Thirty-four nasal swabs, 34 urine and 13 fecal samples were collected from 34 swine farmers who worked in the seven participating farms. The human subjects were advised to collect midstream catch urine samples. Environmental samples included 14 drinking water samples, 14 feed samples and 14 pen swabs were also collected from each farm. Background information of the samples were collected based on questionnaire and observation of the same attending veterinarian. The information such as farm location, hygiene practice, gender of the pigs, body temperature and health condition of the pigs (healthy/unhealthy) were collected and used for statistical analysis. The farm hygiene practices were arbitrarily divided into three categories: HP1 refers to application of fundamental hygiene practice (clean the pens with detergents only). HP2 refers to a better level of hygiene practice, includes cleaning the pens and disinfecting equipment. HP3 refers to the best hygiene practice, includes personal hygiene practice of farmers, foot-dip practices and all above mentioned hygiene practices. Physical examination was performed by the field veterinarian to determine the health status of pigs. Pigs that presented with abnormal clinical signs, behavior and elevated body temperature were categorized as unhealthy. This study was approved by the Animal Care and Use Committee (ACUC), Faculty of Veterinary Medicine, UPM (UPM/IACUC/FYP-AUP-T006/). The human samples collection was approved by Medical Ethics Committee, University Malaya Medical Centre (Ethic committee/IRB reference number: 1010.41), with written informed consent of the human subjects. All swabs were collected using sterile Transwab^®^ Amies Charcoal (Medical Wire & Equipment Co, Corsham, UK). All the samples were transported on ice and processed within 24 h upon collection.

### Bacterial isolation and identification

All samples except fecal samples were enriched in Brain Heart Infusion (BHI) medium containing 6.5% NaCl followed by plating on the CHROMagar Orientation (CHROMagar Company, Paris, France). For fecal samples, one gram of feces was suspended in two ml of 0.85% saline before being streaked onto CHROMagar Orientation.

Approximately 3,450 presumptive colonies were obtained in the initial screening based on the manufacturer’s guidelines. Due to the large numbers of isolates, 10% of turquoise blue colored colonies (approximately 10–15 colonies per plate) were selected for further identification using Vitek-MS (Biomérieux, Marcy I’Etoile, France) according to the manufacturer’s protocol. The *Escherichia coli* (ATCC 8739) strain was used as the control. PCR targeting *sodA* genes was used to confirm the identity of the strains (Jackson, Fedorka-Cray & Barrett, 2004).

### Antibiotic susceptibility test

Antibiotic susceptibility test for non-repeat strains was determined with commercial antimicrobial discs (Oxoid, Basingstoke, United Kingdom) by using the disk diffusion method according to the Clinical and Laboratory Standards Institute guidelines (CLSI, 2015). The bacterial strains were tested against a panel of antimicrobial agents which are commonly prescribed for enterococcal infections and frequently used in the swine farms including ampicillin (AMP, 10 µg), vancomycin (VAN, 30 µg), chloramphenicol (C, 30 µg), tetracycline (TET, 30 µg), ceftiofur (EFT, 30 µg), ciprofloxacin (CIP, 5 µg), erythromycin (E, 15 µg), low-level gentamicin (CN, 10 µg), high-level gentamicin (HLG, 120 µg), linezolid (LZD, 30 µg), penicillin (P, 10U) and teicoplanin (TEC, 30 µg). Strains with zones of inhibition under intermediate and resistant categories were defined as non-susceptible in this study. *Staphylococcus aureus* ATCC 25923 and *E. faecalis* ATCC 29212 were used as control strains. Multidrug resistance of the strain was determined by antibiotics which are not intrinsically resistant by *E. faecalis*, according to the Clinical and Laboratory Standards Institute (CLSI) guidelines.

### PCR detection of antibiotic resistance genes and virulence genes

Detection of antibiotic resistance genes and virulence genes were used to determine the resistance and virulence potential of the strains. Boiled cell lysates were used as DNA templates. Antibiotic resistance and virulence genes were identified by Polymerase Chain Reaction (PCR) using primers and condition as previously described (File S1).

### Biofilm assay

To study the biofilm forming ability, crystal violet assay was performed using the protocol described by Baldassarri et al. (2001) with slight modifications. Biofilm was allowed to grow at 37 °C for 48 h and the optical density (OD) of eluted crystal violet stain was measured at OD_570 nm_. The true OD readings of each strain were acquired after deducting the negative control, which contained only the growth medium. The biofilm forming ability of the studied strains was scored as previously described (Stepanovic et al., 2000). A strong biofilm forming strain VREr5 from Lim, Teh & Thong (2017) was used as a positive control.

### Repetitive extragenic palindromic-PCR (REP-PCR)

REP-PCR was performed using primer (GTG)_5_ as described by Versalovic et al. (1994). DNA extraction was performed using Genomic DNA Mini kit (Yeastern Biotech Co., Taiwan) according to the manufacturer’s guidelines. PCR was carried out in a total volume of 25 µl master mix containing 1X buffer, 2.5 mM of MgCl_2_, 0.8 mg/ml of bovine serum albumin (BSA), 0.1 mM of dNTPs, 1.6 µM of primers and 1.0 U of *Taq* polymerase (Promega, Madison, USA). The PCR protocol involved an initial denaturation of 7 min at 95 °C, followed by 30 cycles of 90 °C for 30 s, 40 °C for 1 min, 65 °C for 8 min and a final elongation of 16 min at 65 °C. The amplicons generated were electrophoresed on a 1.5% agarose gel at 100 V for 6 h. A 1 kb DNA marker (Promega, Madison, WI, USA) was used as the molecular size standard. The gels were stained with Gel-Red and visualized with Gel Doc XR imaging system (Bio-Rad, Hercules, CA, USA). The banding patterns generated were analyzed with BioNumerics 6.0 (Applied Maths, Kortrijk, Belgium). All the PCR fingerprints profiles were assigned an arbitrary designation. The quantitative difference among the profiles was defined by the Dice coefficient, Hierarchical clustering analysis was carried out based on unweighted pair group with arithmetric averages (UPGMA) using 1.5% tolerance. REP-PCR was repeated twice to ensure the reproducibility.

### Pulsed-Field Gel Electrophoresis (PFGE)

PFGE for *Sma*I-digested genomic DNA was performed in a CHEF Mapper (Bio-Rad, Hercules, CA, USA) as described by Turabelidze et al. (2000) with some modification in the DNA preparation. In brief, the bacteria cells were lysed with a combination of lysozyme (2.5 mg/ml) and mutanolysin (1,250 U/ml) for 4 h at 37 °C. Bacterial cells were lysed overnight in cell lysis buffer containing 0.5M EDTA, 1% sarcosyl and 50 µg/ml of proteinase K. The plugs were washed thoroughly with TE buffer. Slices of DNA plugs were digested overnight with *Sma*I at 25 °C and *Xba*I at 37 °C. PFGE was run using pulse times 3.5 s to 25 s for 12 h (block 1, 6V, 120°) and 1 s to 5 s for 10 h (block 2, 6V, 120°). *Xba*I-digested *Salmonella enterica* serovar Braenderup H9812 was used as the size marker. PFGE was repeated once to ensure reproducibility. The PFGE profiles were analyzed by BioNumerics version 6.0 software (Applied Maths, Kortrijk, Belgium). A dendrogram was constructed based on Dice coefficient of similarity (F) and unweighted pair group method with arithmetic mean (UPGMA) algorithm with 1.5% position tolerance.

### Statistical analysis

The environmental factors used for correlation study included sample subjects, sample matrix, sampling region and farm hygiene practice. The association of genotypes with resistotypes, virulotypes, biofilm-forming ability and the environmental factors were determined using distance-based linear model (DistLM) by PRIMER 6 with PERMANOVA add on package (PRIMER-E, UK). The correlation between the resistotypes and virulotypes with other factors were tested with crosstabs/chi-square analysis using SPSS version 21 (IBM SPSS Statistics; IBM corporation, Armonk, NY, USA).

## Results

### Occurrence of *E. faecalis* and *E. faecium*

A total of 289 *Enterococcus* strains (232 from pigs, 54 from farmers and three from farm environments) were isolated. These strains were affiliated with five species, which included *E. faecalis* (*n* = 211)*, E. faecium* (*n* = 42)*, E. hirae* (*n* = 15)*, E. gallinarium* (*n* = 11) and *E. casseliflavus* (*n* = 10). All *E. faecalis* and *E. faecium* strains were confirmed with PCR targeting the *sodA* genes (Jackson, Fedorka-Cray & Barrett, 2004).

**Figure 2:**
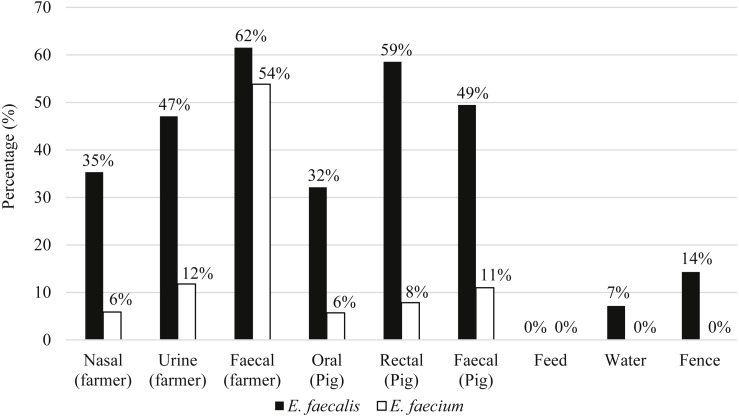
Percentage of distribution of *E. faecalis* and *E. faecium* in each sample matrix.

Eighty-seven pigs and 21 farmers harbored either *E. faecalis*, *E. faecium* or both species of *Enterococcus*. Figure 2 shows the distribution of *E. faecalis* and *E. faecium* in each type of sample matrix. Higher occurrence of enterococci was observed in humans compared to pigs and only three *E. faecalis* strains were isolated from the environmental samples (Fig. 2). *E. faecalis* was isolated from all participating farms but *E. faecium* was restricted to the swine samples collected from farms PF2, PF4, PF5, SF1 and SF2 as well as the human samples from PF1, PF4 and SF1.

### Antimicrobial susceptibility of *E. faecalis* and *E. faecium*

**Figure 3:**
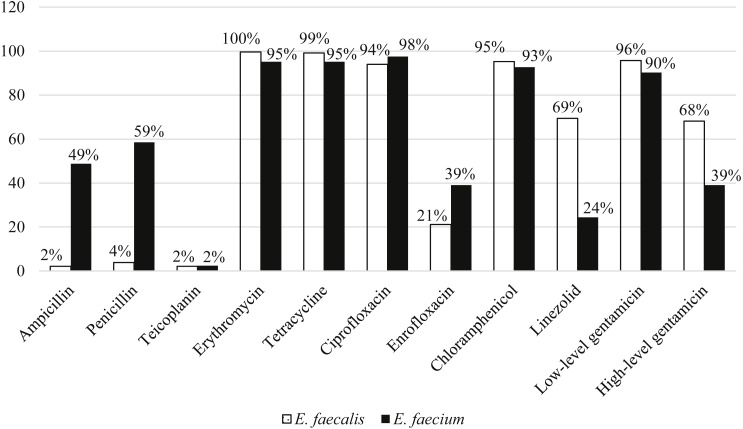
Percentage of antibiotic resistance of *E. faecalis* and *E. faecium* in this study.

Among the 211 *E. faecalis* strains, 98% and 96% of them were susceptible to ampicillin and penicillin, respectively. However, *E. faecium* were more resistant to ampicillin (49%) and penicillin (59%) compared to *E. faecalis*. All the strains were susceptible to vancomycin and only two percent of strains from each species were resistant to teicoplanin. *E. faecalis* (68%) strains were relatively more resistant to high-level gentamicin (120 µg) compared to *E. faecium* (39%). Overall, more than 90% of both species were resistant to erythromycin, tetracycline, ciprofloxacin, chloramphenicol and low-level gentamicin (Fig. 3).

**Table 1:** Resistotypes of *Enterococcus faecium* strains based on the classes of antibiotics tested.

Resistotypes (*n*_P_, *n*_H_, *n*_E_)^a^	Classes of antibiotic resistances^b^											
	C1		C2		C3	C4	C5		C6	C7	C8	
	P	AMP	VAN	TEC	E	TET	CIP	ENR	C	LZD	CN	HLG
R1 (*n*_P_ = 1, *n*_H_ = 0, *n*_E_ = 0)					R	R						
R2 (*n*_P_ = 1, *n*_H_ = 0, *n*_E_ = 0)					R	R			R			
R3 (*n*_P_ = 0, *n*_H_ = 1, *n*_E_ = 0)					R			R	R			
R4^c^(*n*_P_ = 6, *n*_H_ = 0, *n*_E_ = 0)					R	R			R		R	
R5 (*n*_P_ = 1, *n*_H_ = 0, *n*_E_ = 0)					R	R	R		R			
R6 (*n*_P_ = 2, *n*_H_ = 0, *n*_E_ = 0)					R	R	R				R	R
R7 (*n*_P_ = 8, *n*_H_ = 0, *n*_E_ = 1)					R	R	R		R		R	
R8 (*n*_P_ = 2, *n*_H_ = 0, *n*_E_ = 0)					R	R	R		R	R		
R9 (*n*_P_ = 1, *n*_H_ = 1, *n*_E_ = 0)					R	R			R	R	R	
R10 (*n*_P_ = 0, *n*_H_ = 1, *n*_E_ = 0)					R	R	R			R	R	
R11 (*n*_P_ = 0, *n*_H_ = 1, *n*_E_ = 0)					R	R	R	R			R	
R12 (*n*_P_ = 0, *n*_H_ = 2, *n*_E_ = 0)					R	R	R	R	R		R	
R13^c^(*n*_P_ = 24, *n*_H_ = 1, *n*_E_ = 0)					R	R	R		R	R	R	
R14^c^(*n*_P_ = 32, *n*_H_ = 0, *n*_E_ = 0)					R	R	R		R		R	R
R15 (*n*_P_ = 3, *n*_H_ = 2, *n*_E_ = 1)					R	R			R	R	R	R
R16 (*n*_P_ = 0, *n*_H_ = 1, *n*_E_ = 0)						R	R	R	R	R	R	
R17^c^(*n*_P_ = 2, *n*_H_ = 0, *n*_E_ = 0)	R				R	R	R		R		R	
R18^c^(*n*_P_ = 2, *n*_H_ = 0, *n*_E_ = 0)	R				R	R	R	R	R			
R19^c^(*n*_P_ = 7, *n*_H_ = 0, *n*_E_ = 0)					R	R	R	R	R		R	R
R20^c^(*n*_P_ = 61, *n*_H_ = 9, *n*_E_ = 1)					R	R	R		R	R	R	R
R21 (*n*_P_ = 0, *n*_H_ = 1, *n*_E_ = 0)					R		R	R	R	R	R	R
R22 (*n*_P_ = 0, *n*_H_ = 3, *n*_E_ = 0)					R	R	R	R	R	R	R	
R23 (*n*_P_ = 1, *n*_H_ = 0, *n*_E_ = 0)	R				R	R	R		R		R	R
R24 (*n*_P_ = 1, *n*_H_ = 0, *n*_E_ = 0)		R			R	R	R	R	R	R		
R25 (*n*_P_ = 1, *n*_H_ = 0, *n*_E_ = 0)	R				R	R	R		R	R	R	
R26^c^(*n*_P_ = 11, *n*_H_ = 10, *n*_E_ = 0)					R	R	R	R	R	R	R	R
R27 (*n*_P_ = 1, *n*_H_ = 0, *n*_E_ = 0)				R	R	R	R		R	R	R	R
R28 (*n*_P_ = 1, *n*_H_ = 0, *n*_E_ = 0)				R	R	R	R	R		R	R	R
R29^c^(*n*_P_ = 1, *n*_H_ = 0, *n*_E_ = 0)	R	R			R	R	R		R		R	R
R30 (*n*_P_ = 0, *n*_H_ = 1, *n*_E_ = 0)		R			R	R	R	R	R	R	R	
R31 (*n*_P_ = 0, *n*_H_ = 1, *n*_E_ = 0)	R				R	R	R		R	R	R	R
R32 (*n*_P_ = 0, *n*_H_ = 1, *n*_E_ = 0)				R	R	R	R	R	R	R	R	R
R33 (*n*_P_ = 1, *n*_H_ = 0, *n*_E_ = 0)	R	R			R	R	R		R	R	R	R
R34^c^(*n*_P_ = 1, *n*_H_ = 0, *n*_E_ = 0)	R	R			R	R	R	R	R	R	R	R

**Notes.**

a *n*_P_, number of swine isolates; *n*_H_, number of human isolates, *n*_E_: number of environmental isolates.

b C1, penicillin; C2, glycopeptides; C3, macrolides; C4, tetracyclines; C5, fluoroquinolones; C6, phenicols; C7, oxazolidinones; C8, aminoglycosides; AMP, ampicillin; P, penicillin; TEC, teicoplanin; E, erythromycin; TET, tetracycline; CIP, ciprofloxacin; ENR, enrofloxacin; C, chloramphenicol; LZD, linezolid; CN, gentamicin; HLG, high-level gentamicin; R, resistant.

c Resistotypes shared by both *E. faecalis* and *E. faecium*.

Ninety-eight percent of the strains were MDR (non-susceptible to at least 4 classes of antibiotics) and were resistant to chloramphenicol, tetracycline, ciprofloxacin, gentamicin and erythromycin. There were 47 resistotypes observed in this study. Thirty-four and 23 resistotypes were observed in *E. faecalis* (Table 1) and *E. faecium* (Table 2)*,* respectively. Ten resistotypes were shared by both species of bacteria. The resistance patterns observed in swine strains were more diverse than human strains. Resistotypes R20 and R26 were most prevalent among the *E. faecalis* strains and were resistant to erythromycin, tetracycline, ciprofloxacin, chloramphenicol and gentamicin. Resistance genes detection by PCR showed that all the strains tested in study harbored at least one of the antibiotic resistance genes tested. For the genes encoding for erythromycin resistance, *erm*B was the most prevalent compared to *ermA* and *msr.* A total of 96% and 95% of *E. faecalis* and *E. faecium* strains, respectively, harbored *erm*B. Besides, 90% of both *E. faecalis* and *E. faecium* strains possessed *tet*L and 98% of *E. faecalis* and 95% of *E. faecium* contained *tet*M gene encoding tetracycline resistance. At least one aminoglucoside resistance gene was present in 89% of the *E. faecalis* and 83% of the *E. faecium* strains. Among the six aminoglycoside resistance genes, *aac(6′)-Ie-aph(2″)-Ia* and *aph(3′)-IIIa* were detected in most of the strains (64% with *aac(6′)-Ie-aph(2″)-Ia* and 87% with *aph(3′)-IIIa*). Notably, four strains harbored vancomycin resistance genes (*van* B and *van* C1) but none were resistant to vancomycin based on disk diffusion method.

**Table 2:** Resistotypes of *Enterococcus faecalis* strains based on the classes of antibiotics tested.

Resistotypes^a^	Classes of antibiotic resistances^b^											
	C1		C2		C3	C4	C5		C6	C7	C8	
	P	AMP	VAN	TEC	E	TET	CIP	ENR	C	LZD	CN	HLG
R4^c^(*n*_P_ = 0, *n*_H_ = 1, *n*_E_ = 0)					R	R			R		R	
R13^c^(*n*_P_ = 2, *n*_H_ = 0, *n*_E_ = 0)					R	R	R		R	R	R	
R14^c^(*n*_P_ = 1, *n*_H_ = 0, *n*_E_ = 0)					R	R	R		R		R	R
R17^c^(*n*_P_ = 2, *n*_H_ = 0, *n*_E_ = 0)	R				R	R	R		R		R	
R18^c^(*n*_P_ = 0, *n*_H_ = 1, *n*_E_ = 0)	R				R	R	R	R	R			
R19^c^(*n*_P_ = 2, *n*_H_ = 0, *n*_E_ = 0)					R	R	R	R	R		R	R
R20^c^(*n*_P_ = 1, *n*_H_ = 0, *n*_E_ = 0)					R	R	R		R	R	R	R
R26^c^(*n*_P_ = 0, *n*_H_ = 4, *n*_E_ = 0)					R	R	R	R	R	R	R	R
R29^c^(*n*_P_ = 2, *n*_H_ = 0, *n*_E_ = 0)	R	R			R	R	R		R		R	R
R34^c^(*n*_P_ = 2, *n*_H_ = 0, *n*_E_ = 0)	R	R			R	R	R	R	R	R	R	R
R35 (*n*_P_ = 0, *n*_H_ = 2, *n*_E_ = 0)					R		R				R	
R36 (*n*_P_ = 1, *n*_H_ = 2, *n*_E_ = 0)						R	R		R		R	
R37 (*n*_P_ = 0, *n*_H_ = 1, *n*_E_ = 0)					R	R	R	R	R	R		
R38 (*n*_P_ = 1, *n*_H_ = 0, *n*_E_ = 0)		R			R	R	R		R		R	
R39 (*n*_P_ = 2, *n*_H_ = 0, *n*_E_ = 0)	R	R			R	R	R		R			
R40 (*n*_P_ = 1, *n*_H_ = 0, *n*_E_ = 0)	R				R	R	R		R		R	R
R41 (*n*_P_ = 1, *n*_H_ = 0, *n*_E_ = 0)	R				R	R	R	R			R	R
R42 (*n*_P_ = 0, *n*_H_ = 1, *n*_E_ = 0)	R				R	R	R	R	R		R	
R43 (*n*_P_ = 0, *n*_H_ = 1, *n*_E_ = 0)		R			R	R	R	R	R		R	
R44 (*n*_P_ = 5, *n*_H_ = 0, *n*_E_ = 0)	R	R			R	R	R		R		R	
R45 (*n*_P_ = 1, *n*_H_ = 0, *n*_E_ = 0)	R	R		R	R	R	R		R		R	
R46 (*n*_P_ = 3, *n*_H_ = 0, *n*_E_ = 0)	R	R			R	R	R	R	R		R	
R47 (*n*_P_ = 2, *n*_H_ = 0, *n*_E_ = 0)	R	R			R	R	R	R	R		R	R

**Notes.**

a *n*_P_, number of swine isolates; *n*_H_, number of human isolates; *n*_E_, number of environmental isolates.

b C1, penicillin; C2, glycopeptides; C3, macrolides; C4, tetracyclines; C5, fluoroquinolones; C6, phenicols; C7, oxazolidinones; C8, aminoglycosides; AMP, ampicillin; P, penicillin; TEC, teicoplanin; E, erythromycin; TET, tetracycline; CIP, ciprofloxacin; ENR, enrofloxacin; C, chloramphenicol; LZD, linezolid; CN, gentamicin; HLG, high-level gentamicin; R, resistant.

c Resistotypes shared by both *E. faecalis* and *E. faecium*.

### Virulotyping

A total of 99% and 57% of the *E. faecalis* and *E. faecium* strains, respectively, harbored at least one of the five virulence genes screened in this study. *efa* was the most prevalent virulence gene detected in *E. faecalis* strains (90%), followed by *asa*I (68%), *gel*E (63%), *esp* (40%), *cyl* (40%) and *ace* (15%)*.* In contrast*, asa*I was most prevalent in *E. faecium* (43%). Other virulence genes including *esp*, *gel*E, *efa* and *cyl* were also present in 21%, 19%, 17% and 14% of *E. faecium,* respectively. None of the strains harbored *hyl* gene.

### Biofilm assay

Overall, about 62% of the strains from pigs and humans are capable of producing biofilms. Among the 142 biofilm-forming *E. faecalis* strains, 37%, 34% and 29% were weak, moderate and strong biofilm formers, respectively. Eight *E. faecium* strains formed weak biofilm and notably, half of them were isolated from human urine samples. All three environmental strains could form strong biofilm.

### Genotypic characterization by REP-PCR and PFGE

REP-PCR subtyped the 211 non-repeat *E. faecalis* strains into 145 REP-profiles with 9 to 21 DNA fragments ranging in size from 500 to 6,000 bp. Reproducible patterns were observed. Based on 85% similarity, the *E. faecalis* strains were grouped into 24 clusters and 13 unique patterns (Files S2). From the dendrogram, the strains isolated from different samples matrix (oral swabs, nasal swabs, rectal swabs, urine and fecal samples) were grouped in the same cluster. However, distinct clustering was observed for strains originated from different regions (northern and central region of Peninsular Malaysia). Strains isolated from both regions were observed in Clusters C4, C5 and C9. Overall, REP-patterns of strains from central region were host-specific (Clusters C2, C19, C23 and C24 comprised of strains isolated from humans and Clusters C7, C13, C20, C21 and C22 comprised of strains isolated from pigs). Three environmental *E. faecalis* strains were clustered with the swine and human strains. For instance, Enfs99 recovered from a pen swab in PF1 was clustered with human nasal strain from the same farm in C15 (Files S2). Enfs103 isolated from a pen swab was 95% similar to the swine oral strains isolated from the same farm, PF1. Enfs98 from the water sample of PF3 formed cluster C10 with the swine fecal strains of the same farm.

*E. faecium* strains were grouped into 11 clusters and 11 unique patterns based on 85% similarity of the REP-profiles (Files S3). Similar to *E. faecalis*, the strains were regional specific. Strains in Clusters C1, C2, C3 and C5 were from the northern region and Clusters C4, C6 to C11 were made up of strains isolated from the central region only. The *E. faecium* strains from the central region were also host specific. Human strains were grouped in Clusters C4 and C9 while swine-related strains were grouped in Clusters C6, C7, C8, C10 and C11. In contrast, Cluster C2 consisted of both human- and swine-related strains isolated from the northern region.

Similar clustering and clonality of the strains were observed based on PFGE. PFGE generated 126 reproducible pulsotypes for *E. faecalis* with 12 to 26 bands and grouped into 27 clusters and 11 unique patterns based on 85% similarity (Files S4). Major clusters were represented by Clusters C15, C16 and C22, comprised of strains isolated from both humans and pigs. However, regional- and host-specific clusters were also observed in the dendrogram (Files S4). Meanwhile, 35 pulsotypes of *E. faecium* strains were grouped into 11 clusters and 6 unique patterns (Files S5). All the clusters were regional specific and Cluster C2 was made up of strains from both pigs and humans.

### Antibiotic resistance profiles of *E. faecalis* and *E. faecium* strains

Correlations between the antibiotic resistance of *E. faecalis* and *E. faecium* strains with different factors were determined by chi-squared test (Table 3). Ampicillin resistance of *E. faecalis* was correlated to the sampling region and biofilm-forming ability. High-level gentamicin resistance of *E. faecalis* was correlated with farm locality, sample matrix, farm hygiene practice and the biofilm-forming ability of the strains. In contrast, ampicillin resistance of *E. faecium* was correlated with all the environmental factors. Penicillin and high-level gentamicin resistance were correlated with the biofilm-forming ability of the strains. Strains isolated from different regions possessed different ampicillin and erythromycin resistance. The farm hygiene practice was found to correlate with the strains’ resistance towards ampicillin, penicillin, tetracycline and enrofloxacin (Table 3).

**Table 3:** Significance of correlation between the antibiotic resistances of different samples classification based of Chi-squared test.

	*P*-values (*P* < 0.05 = rejectnull)^a^^,^^b^										
Groups^c^	AMP 10	P 10	TEC 30	E 5	TET 30	CIP 5	ENR 5	C 30	LZD 30	CN 10	CN 120
*E. faecalis*:											
Host	x	x	x	x	0.007	x	x	x	0.014	x	x
Biofilm	0.002	x	x	x	x	x	x	x	x	0.031	0.023
Matrix	x	x	x	0.001	0.010	0.006	x	x	0.006	x	0.018
Region	0.006	x	x	x	x	x	x	x	x	x	0.001
Hygiene	x	x	x	x	x	x	x	x	x	x	0.010
Virulotypes	x	0.030	x	x	x	x	x	0.001	0.018	x	x
*E. faecium*:											
Host	0.002	0.001	x	x	x	x	x	x	x	x	x
Biofilm	x	0.015	x	x	x	x	x	x	x	x	0.038
Matrix	0.033	x	x	x	x	x	0.010	x	0.007	x	0.006
Region	0.023	x	x	0.040	x	x	x	x	x	x	x
Hygiene	x	0.032	x	x	0.035	x	0.004	x	x	x	x
Virulotypes	x	0.030	x	x	x	x	x	0.001	0.018	x	x

**Notes.**

a AMP, ampicillin; P, penicillin; TEC, teicoplanin; E, erythromycin; TET, tetracycline; ENR, enrofloxacin; CIP, ciprofloxacin; C, chloramphenicol; LZD, linezolid; CN, gentamicin.

b x, No significant correlation.

c Subject, human host and swine host; Biofilm refers to biofilm former and non-former; Matrix, oral, rectal, nasal, urine and fecal; Region, northern region and central region; hygiene, HP1, HP2, HP3.

The presence of *ace* gene in *E. faecalis* strains was dependent on the sample matrix and sampling region while the *efa* gene was correlated with sampling region, sample matrix, farm hygiene practice and resistotypes (Table 4). On the other hand, the presence of *efa* gene in *E. faecium* was correlated with the sampling region and virulotypes of the strains.

**Table 4:** Correlation between the presence of virulence genes with environmental factors, virulotypes and resistotypes.

	*P*-values (*P* < 0.05 = rejectnull)^a^^,^^b^					
Groups^c^	*ace*	*efa*	*gel*E	*asa*	*esp*	*cyl*
*E. faecalis*:						
Host	x	x	x	x	x	x
Biofilm	x	x	x	x	x	x
Matrix	0.034	0.019	x	x	x	x
Region	0.049	0.001	0.021	x	0.004	x
Hygiene	x	0.033	x	x	0.006	x
Resistotypes	x	0.037	0.022	x	x	x
Virulotypes	x	x	x	x	x	x
*E. faecium*:						
Host	x	x	x	x	x	x
Biofilm	x	x	x	x	x	x
Matrix	x	x	x	x	x	x
Region	x	0.038	x	x	x	x
Hygiene	x	x	x	x	x	x
Resistotypes	x	x	x	x	0.009	x
Virulotypes	x	0.020	x	x	x	x

**Notes.**

a *ace*, collagen binding cell wall protein; *efa*, endocarditis specific antigen; *gel*E, gelatinase; *asa*, aggregation substance; *esp*, enterococcal surface protein; *cyl*, cytolysin

b x, No significant correlation.

c Subject: human host and swine host; Biofilm refers to biofilm former and non-former; Matrix, oral, rectal, nasal, urine and fecal; Region, northern region and central region; Hygiene, HP1, HP2, HP3.

### Distribution of the *E. faecalis* and *E. faecium* strains across locations, host and sample type, and its relationship to the biofilm-forming ability

To determine the association between the genotypes, resistotypes, virulotypes and different environmental factors, a composite similarity matrix of the two typing methods was used to perform statistical analysis on the correlation between the genotype of the strains and different factors. Based on the DistLM results, the DNA fingerprints of strains isolated from both pigs and humans were significantly different among each other (Pseudo-*F* = 2.5204, *P* = 0.020). We further evaluated the factors which might affect the genotypes of the strains such as sample matrix, sampling region, farm hygiene practice, biofilm-forming ability as well as the resistotypes and virulotypes of the strains (Table 5). For the *E. faecalis* isolated from pigs, a significant correlation was detected for all tested factors except biofilm-forming ability while for human strains, no association was found between the genotypic patterns in relation to farm hygiene practice, sample matrix and biofilm-forming ability. Conversely, *E. faecium* isolated from swine samples were significantly associated with the sampling region and hygiene practice. The strains also exhibited significant genotypic difference in association with resistotypes and virulotypes. *E. faecium* strains isolated from human samples was the only group of strains which was significantly correlated to biofilm-forming ability and similar to *E. faecalis*, they were unaffected by the farm hygiene practice. Overall, the statistical analysis supported the finding that the strains isolated in this study are host and regional specific. Strong correlations were also found between the genotypes and the resistance and virulence profiles of the strains.

**Table 5:** Correlation between environmental factors, virulotypes and resistotypes with composition phylogenetic relationship inferred using REP-PCR and PFGE.

Species	Subjects	Factors^a^	Composite of PFGE and REP-PCR	
			Pseudo *F*	*P*
*E. faecalis*	Pigs	Region	26.229	0.001
		Hygiene practice	24.392	0.001
		Sample matrix	5.6083	0.001
		Resistotypes	2.8807	0.014
		Virulotypes	4.7217	0.001
		Biofilm	1.3114	0.244
	Humans	Region	4.9953	0.001
		Hygiene practice	0.5268	0.767
		Sample matrix	1.7178	0.126
		Resistotypes	2.3653	0.048
		Virulotypes	6.0838	0.001
		Biofilm	1.1187	0.361
*E. faecium*	Pigs	Region	9.3722	0.001
		Hygiene practice	7.7394	0.001
		Sample matrix	0.2937	0.902
		Resistotypes	1.6440	0.159
		Virulotypes	4.5310	0.006
		Biofilm	2.2920	0.055
	Humans	Region	6.7071	0.001
		Hygiene practice	2.8370	0.050
		Sample matrix	4.7811	0.010
		Resistotypes	2.6958	0.046
		Virulotypes	2.9966	0.037
		Biofilm	4.4749	0.007

**Notes.**

a Subject, human host and swine host; Biofilm refers to biofilm former and non-former; Matrix, oral, rectal, nasal, urine and fecal; Region: northern region and central region; Hygiene, HP1, HP2, HP3.

## Discussion

More than 90% of the enterococci studied were MDR. Traditionally, the first line treatment for enterococcal infection is a combination of cell wall-active agents and aminoglycosides (Kristich, Rice & Arias, 2014). The occurrence of resistance to high-level gentamicin compromises the efficacy of this drug and the increasing MDR status further limits the antimicrobial therapy. The degree of antibiotic resistance in the herd is strongly correlated with the antibiotic usage as the routine application of antibiotics serves as a selective pressure for resistant bacteria (Woolhouse et al., 2015; Benjamin, Bruce & Lance, 2017). According to the National Pharmaceutical Control Bureau of Ministry of Health, Malaysia, there are 97 antimicrobials registered for the use in livestock. A majority of these antimicrobials are used in poultry and swine farms despite the fact that some of these drugs are classified as Critically Important Antimicrobials by WHO (Health Action International Asia Pacific, 2013). In Malaysia, the Department of Veterinary Services (DVS) is responsible for certification, inspection, accreditation and implementation of legislation to ensure the production of quality livestock. They also ensure that the use of antibiotics is in accordance with the Feed Act 2009 of Malaysia. Taking into consideration the effect of antimicrobial resistance in public health and livestock industry, the Malaysian government has embarked multiple strategies against the antimicrobial resistance threat, which included increase public awareness and education on appropriate use of antibiotics, expedite surveillance and research, and improve infection prevention and control (MOH Malaysia, 2017). The unregulated use of antibiotics in swine husbandry, the co-transfer of resistance genes between enterococcal strains from different origins might have contributed to the high prevalence of resistant strains in this study (Silveira et al., 2013). The strains isolated in this study were mainly resistant to chloramphenicol, tetracycline, ciprofloxacin, gentamicin and erythromycin. Similar results were also observed in study on swine meat chain (Rizzotti, Rossi & Torriani, 2016). *E. faecium* was more resistant to penicillin and ampicillin while *E. faecalis* was more resistant to aminoglycosides. Larsen et al. (2011) reported that porcine-origin *E. faecalis* strains were genetically related to the strains isolated from infective endocarditis patients, suggesting that pigs can be a reservoir of human pathogens. Farm workers, veterinarians and those who are in close contact with the animals are at a higher risk of being colonized or infected by resistant bacteria harbored by the animal hosts. The resistance and virulence genes can also be transferred to other pathogenic bacteria such as *Staphylococcus aureus,* causing severe bacteremia (Kristich, Rice & Arias, 2014; Health Action International Asia Pacific, 2013). Strains with a particular resistance phenotype harbored the corresponding antibiotic resistance genes. Notably, *E. faecalis* strains showing high-level gentamicin resistance harbored two resistance genes, which are the *aac(6′)-Ie-aph(2″)-Ia* and *aph(3′)-IIIa*. Strains with only one aminoglycoside resistance gene were either susceptible or resistant to low-level gentamicin only. However, this trend was only observed in the *E. faecalis* strains. Vancomycin-resistant enterococcus (VRE) have been previously reported from swine farms located in Selangor, Perak, Johor and Penang (Getachew et al., 2010; Getachew et al., 2012; Getachew et al., 2013). However, these studies mainly focused on vancomycin resistance phenotype as VRE strains are important nosocomial pathogens and molecular evidence have also suggested that animals are reservoir of VRE (Courvalin, 2005). Although VRE was not found in our study, our findings showed that enterococcal strains found in the swine farms were multidrug-resistant. This indicates the importance of screening other antibiotics apart from vancomycin.

Biofilms facilitate antibiotic resistance and host colonization of pathogens (Lebreton, Willems & Gilmore, 2014). Our results showed that *E. faecalis* has a higher potential to form biofilm than *E. faecium*. Biofilm-forming *E. faecalis* found in this study possessed higher rates of resistance towards ampicillin, penicillin, chloramphenicol and high-level gentamicin. A mature enterococcal biofilm contains more bacteria and is more tolerant to antibiotics (Holmberg & Rasmussen, 2016). This probably explains the higher infection rate of *E. faecalis* compared to *E. faecium,* because the biofilm-forming *E. faecalis* strains are more persistent than *E. faecium*. Toledo-arana et al. (2001) reported that *esp* gene is involved in the biofilm formation of enterococcal strains*.* However, such correlation was not found in our study. In contrast, *efa* and *gel*E were found to have significant correlation with the resistotypes of *E. faecalis* strains.

The statistical analysis of the composite genotypic data showed that *E. faecalis* and *E. faecium* were significantly grouped according to their hosts (humans vs. pig), suggesting a host specific distribution. However, there are exceptions. Certain *E. faecalis* strains isolated from different hosts with high percentage of similarity (>90%) were observed (Files S2 and S4). For instance, Enfs114 from nasal swab of a farmer and Enfs66 from a swine oral swab from PF4 possessed 100% and 91% similarity in REP and PFGE banding patterns, respectively. This result is consistent with the report by Freitas et al. (2011), which showed that certain *E. faecalis* and *E. faecium* strains in humans and pigs were indistinguishable by PFGE and MLST. It is also noteworthy that the strains isolated from swine oral and human nasal swabs were highly similar with the environmental strains, indicating a potential of inter-host dispersal of the enterococci strains, as strains could be transmitted from one host to another host through the environment. Detection of regional-specific strains could be explained by different swine farm management implemented in each farm as different disinfection and antibiotic exposure could act as a selective pressure.

## Conclusion

This study showed a high occurrence of MDR *E. faecalis* and *E. faecium* in the studied swine farms. MDR Enterococci strains from livestock industry harboring various antibiotic resistance and virulence genes pose a significant health risk to the public. Ubiquitous strains shared by different hosts were identified although majority of the strains were regional- and host-specific. A more stringent regulation is needed to ensure the proper use of antibiotics in swine husbandry to reduce the wide spread of MDR strains.

##  Supplemental Information

10.7717/peerj.5353/supp-1 File S1 Primers used for resistance and virulence genes detection

10.7717/peerj.5353/supp-2 File S2 Dendrogram of *E. faecalis* based on REP-PCR

10.7717/peerj.5353/supp-3 File S3 Dendrogram of *E. faecium* based on REP-PCR

10.7717/peerj.5353/supp-4 File S4 Dendrogram of *E. faecalis* based on PFGE

10.7717/peerj.5353/supp-5 File S5 Dendrogram of *E. faecium* based on PFGE

10.7717/peerj.5353/supp-6 File S6 Raw data

## Additional Information and Declarations

### Field Study Permissions

The following information was supplied relating to field study approvals (i.e., approving body and any reference numbers): This study was approved by the Animal Care and Use Committee (ACUC), Faculty of Veterinary Medicine, UPM (UPM/IACUC/FYP-AUP-T006/2013).

### Data Availability

The following information was supplied regarding data availability: The raw data are provided in the Supplemental Files.
