# Supplementary material for: Occurrence of virulent multidrug-resistant Enterococcus faecalis and Enterococcus faecium in the pigs, farmers and farm environments in Malaysia
Source: PeerJ. 2018 Aug 6;6:e5353. doi: 10.7717/peerj.5353 (PMC6084283; doi:10.7717/peerj.5353)
Supplement: File S1 [file peerj-06-5353-s001.pdf]

Occurrence of virulent multidrug-resistant *Enterococcus faecalis and Enterococcus faecium* in the pigs, farmers and farm environments

Journal: Tropical Animal Health and Production

Shiang Chiet Tan[a], Chun Wie Chong[b], Cindy Shuan Ju Teh[c], Peck Toung Ooi[d], Kwai Lin Thong[a]*

[a] Institute of Biological Science, Faculty of Science, University of Malaya, Kuala Lumpur, Malaysia

[b] Department of Life Science, School of Pharmacy, International Medical University, Kuala Lumpur, Malaysia

[c] Department of Medical Microbiology, Faculty of Medicine, University of Malaya, Kuala Lumpur, Malaysia

[d] Department of Veterinary Clinical Studies, Faculty of Veterinary Medicine, University Putra Malaysia, Serdang, Malaysia

* Corresponding author:

Kwai Lin Thong[a]

Email: thongkl@um.edu.my, Tel: +60379674437

Supplementary Table 1. Primers used for resistance and virulence genes detection

No.	Primer name	Primer sequences	Size	Reference
1	*esp*	TTACCAAGATGGTTCTGTAGGCAC<br>CCAAGTATACTTAGCATCTTTTGG	913	Shankar et al., 1999
2	*cyl*	ACTCGGGGATTGATAGGC<br>GCTGCTAAAGCTGCGCTT	688	Vankerckhoven et al., 2004
3	*hyl*	ACAGAAGAGCTGCAGGAAATG<br>GACTGACGTCCAAGTTTCCAA	276	Vankerckhoven et al., 2004
4	*ace*	AAAGTAGAATTAGATCCACAC<br>TCTATCACATTCGGTTGCG	320	Mannu et al., 2003
5	*gel*E	AGTTCATGTCTATTTTCTTCAC<br>CTTCATTATTTACACGTTTG	402	Mannu et al., 2003
6	*efa*A	CGTGAGAAAGAAATGGAGGA<br>CTACTAACACGTCACGAATG	499	Mannu et al., 2003
7	*tet*M	ACAGAAAGCTTATTATATAAC<br>TGGCGTGTCTATGATGTTCAC	171	Aminov et al., 2001
8	*tet*L	GTMGTTGCGCGCTATATTCC<br>TGAAMGRWAGCCCACCTAA	696	Gever et al., 2003
9	*erm*A	TCTAAAAAGCATGTAAAAGAA<br>CTTCGATAGTTTATTAATATTAGT	645	Dutka-Malen et al., 1995
10	*erm*B	GAAAAGGTACTCAACCAAATA<br>AGTAACGGTACTTAAATTGTTTAC	639	Dutka-Malen et al., 1995
11	*msr*A/B	GCAAATGGTGTAGGTAAGACAACT<br>ATCATGTGATGTAAACAAAAT	399	Dutka-Malen et al., 1995
12	*aac(6')-Ie-aph(2")-Ia*	CAGAGCCTTGGGAAGATGAAG<br>CCTCGTGTAATTCATGTTCTGGC	348	Vakulenko et al., 2003
13	*aph(2")-Ib*	CTTGGACGCTGAGATATATGAGCAC<br>GTTTGTAGCAATTCAGAAACACCCTT	867	Vakulenko et al., 2003
14	*aph(2")-Ic*	CCACAATGATAATGACTCAGTTCCC<br>CCACAGCTTCCGATAGCAAGAG	444	Vakulenko et al., 2003
15	*aph(2")-Id*	GTGGTTTTTACAGGAATGCCATC<br>CCCTCTTCATACCAATCCATATAAC	641	Vakulenko et al., 2003
16	*aph(3')-IIIa*	GGCTAAAATGAGAATATCACCGG<br>CTTTAAAAAATCATACAGCTCGCG	523	Vakulenko et al., 2003
17	*ant(4')-Ia*	CAAACTGCTAAATCGGTAGAAGCC<br>GGAAAGTTGACCAGACATTACGAACT	294	Vakulenko et al., 2003
18	*van*A	CATGAATAGAATAAAAGTTGCAATA<br>CCCCTTTAACGCTAATACGATCAA	1,030	Clark et al., 1993
19	*van*B	GTGACAAACCGGAGGCGAGGA<br>CCGCCATCCTCCTGCAAAAAA	433	Clark et al., 1993
20	*van*C1	GGTATCAAGGAAACCTC<br>CTTCCGCCATCATAGCT	822	Dutka-Malen et al., 1995

21	*van*C2/C3	CGGGGAAGATGGCAGTAT<br>CGCAGGGACGGTGATTTT	484	Satake et al., 1997
