# Supplementary material for: Occurrence of virulent multidrug-resistant Enterococcus faecalis and Enterococcus faecium in the pigs, farmers and farm environments in Malaysia
Source: PeerJ. 2018 Aug 6;6:e5353. doi: 10.7717/peerj.5353 (PMC6084283; doi:10.7717/peerj.5353)
Supplement: File S3 [file peerj-06-5353-s003.pdf]

Occurrence of virulent multidrug-resistant *Enterococcus faecalis and Enterococcus faecium* in the pigs, farmers and farm environments

Journal: Tropical Animal Health and Production

Shiang Chiet Tan[a], Chun Wie Chong[b], Cindy Shuan Ju Teh[c], Peck Toung Ooi[d], Kwai Lin Thong[a]*

[a] Institute of Biological Science, Faculty of Science, University of Malaya, Kuala Lumpur, Malaysia

[b] Department of Life Science, School of Pharmacy, International Medical University, Kuala Lumpur, Malaysia

[c] Department of Medical Microbiology, Faculty of Medicine, University of Malaya, Kuala Lumpur, Malaysia

[d] Department of Veterinary Clinical Studies, Faculty of Veterinary Medicine, University Putra Malaysia, Serdang, Malaysia

* Corresponding author:

Kwai Lin Thong[a]

Email: thongkl@um.edu.my, Tel: +60379674437

Supplementary Figure A.2. Dendrogram of *E. faecium* based on REP-PCR

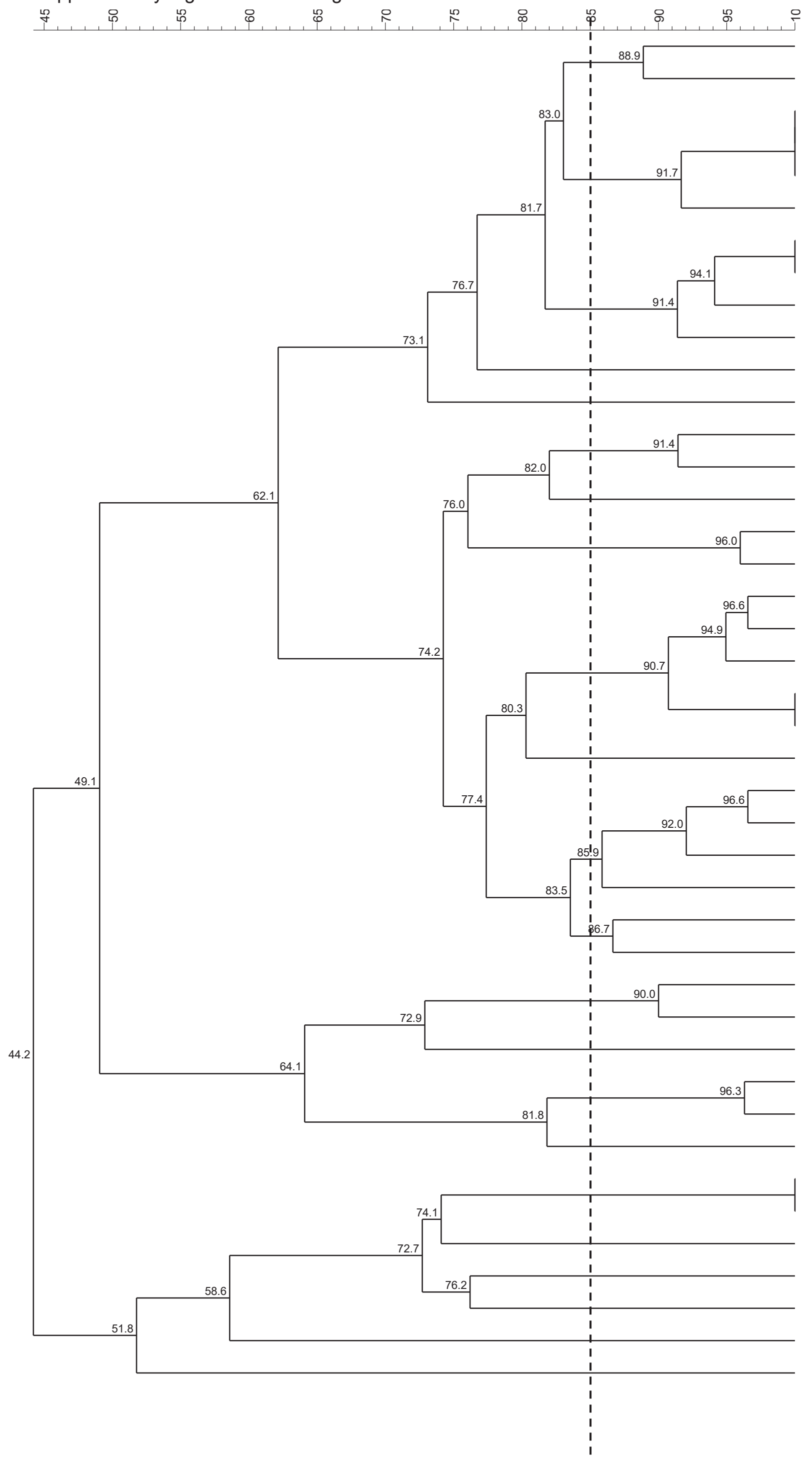

Strain code	Sample Matrix	Sample Subject	Farm	Antibiotic resistance profiles	Biofilm Formation	
Enfm12	Fecal	Human	1	E15, CIP5, CN10	N	C1
Enfm15	Fecal	Human	1	E15, CIP5, CN10	N	
Enfm19	Fecal	Human	4	TET30, CIP5, C30, CN10	N	C2
Enfm20	Fecal	Human	4	TET30, CIP5, C30, CN10	F	
Enfm22	Rectal	Pig	2	TET30, CIP5, C30, CN10	N	
Enfm21	Fecal	Human	1	E15, TET30, C30, CN10	N	
Enfm04	Fecal	Pig	2	E15, TET30, CIP5, C30, LZD30, CN10	N	C3
Enfm06	Fecal	Pig	2	E15, TET30, CIP5, C30, CN10, CN120	N	
Enfm23	Rectal	Pig	2	AMP10, E15, TET30, CIP5, C30, CN10	N	
Enfm05	Fecal	Pig	2	E15, TET30, CIP5, C30, LZD30, CN10	N	
Enfm14	Rectal	Pig	5	P10, E15, TET30, CIP5, C30, CN10	N	
Enfm24	Rectal	Pig	4	P10, E15, TET30, CIP5, C30, CN10, CN120	N	
P.En096	Nasal	Human	6	P10, E15, TET30, CIP5, ENR5, C30	N	C4
P.En097	Nasal	Human	6	P10, E15, TET30, CIP5, ENR5, C30, CN10	N	
Enfm09	Oral	Pig	4	P10, E15, TET30, CIP5, C30, CN10	N	
Enfm07	Oral	Pig	2	AMP10, P10, E15, TET30, CIP5, C30, CN10	N	C5
Enfm08	Oral	Pig	2	AMP10, P10, E15, TET30, CIP5, C30, CN10, CN120	N	
P.En238	Fecal	Pig	6	AMP10, P10, E15, TET30, CIP5, ENR5, C30, CN10	N	C6
P.En239	Fecal	Pig	6	AMP10, P10, TEC30, E15, TET30, CIP5, C30, CN10	N	
P.En240	Fecal	Pig	6	AMP10, P10, E15, TET30, CIP5, ENR5, C30, LZD30, CN10, CN120	N	
P.En066	Rectal	Pig	6	AMP10, P10, E15, TET30, CIP5, C30, CN10	N	
P.En067	Rectal	Pig	6	AMP10, P10, E15, TET30, CIP5, ENR5, C30, CN10	N	
P.En045	Oral	Pig	6	AMP10, P10, E15, TET30, CIP5, ENR5, C30, CN10	N	C7
P.En059	Rectal	Pig	6	AMP10, P10, E15, TET30, CIP5, C30	N	
P.En060	Oral	Pig	6	AMP10, P10, E15, TET30, CIP5, C30, CN10	N	
P.En085	Rectal	Pig	6	AMP10, P10, E15, TET30, CIP5, C30, CN10	N	
P.En065	Oral	Pig	6	AMP10, P10, E15, TET30, CIP5, C30, CN10, CN120	N	
P.En023	Oral	Pig	6	P10, E15, TET30, CIP5, ENR5, CN10, CN120	N	C8
P.En087	Oral	Pig	6	AMP10, P10, E15, TET30, CIP5, ENR5, C30, CN10, CN120	N	
P.En272	Urine	Human	6	E15, TET30, CIP5, ENR5, C30, LZD30, CN10, CN120	F	C9
P.En274	Urine	Human	6	E15, TET30, CIP5, ENR5, C30, LZD30, CN10, CN120	F	
P.En277	Urine	Human	6	E15, TET30, CIP5, ENR5, C30, LZD30, CN10, CN120	F	
P.En233	Fecal	Pig	6	E15, TET30, CIP5, ENR5, C30, CN10, CN120	F	C10
P.En234	Fecal	Pig	6	E15, TET30, CIP5, ENR5, C30, CN10, CN120	F	
P.En269	Urine	Human	6	E15, TET30, CIP5, ENR5, C30, LZD30, CN10, CN120	F	
P.En209	Rectal	Pig	7	AMP10, P10, E15, TET30, CIP5, C30	F	C11
P.En210	Rectal	Pig	7	AMP10, P10, E15, TET30, CIP5, C30, CN10	N	
P.En252	Fecal	Pig	7	AMP10, P10, E15, TET30, CIP5, ENR5, C30, LZD30, CN10, CN120	N	
P.En183	Rectal	Pig	7	E15, TET30, CIP5, C30, LZD30, CN10, CN120	N	
P.En241	Fecal	Pig	6	AMP10, P10, E15, TET30, CIP5, ENR5, C30, CN10, CN120	N	
P.En268	Fecal	Human	6	AMP10, E15, TET30, CIP5, ENR5, C30, CN10	N	
P.En264	Fecal	Human	6	E15, TET30, CIP5, ENR5, C30, LZD30	N	
