# Supplementary material for: Occurrence of virulent multidrug-resistant Enterococcus faecalis and Enterococcus faecium in the pigs, farmers and farm environments in Malaysia
Source: PeerJ. 2018 Aug 6;6:e5353. doi: 10.7717/peerj.5353 (PMC6084283; doi:10.7717/peerj.5353)
Supplement: File S4 [file peerj-06-5353-s004.pdf]

Occurrence of virulent multidrug-resistant *Enterococcus faecalis and Enterococcus faecium* in the pigs, farmers and farm environments

Journal: Tropical Animal Health and Production

Shiang Chiet Tan[a], Chun Wie Chong[b], Cindy Shuan Ju Teh[c], Peck Toung Ooi[d], Kwai Lin Thong[a]*

[a] Institute of Biological Science, Faculty of Science, University of Malaya, Kuala Lumpur, Malaysia

[b] Department of Life Science, School of Pharmacy, International Medical University, Kuala Lumpur, Malaysia

[c] Department of Medical Microbiology, Faculty of Medicine, University of Malaya, Kuala Lumpur, Malaysia

[d] Department of Veterinary Clinical Studies, Faculty of Veterinary Medicine, University Putra Malaysia, Serdang, Malaysia

* Corresponding author:

Kwai Lin Thong[a]

Email: thongkl@um.edu.my, Tel: +60379674437

Supplementary Figure A.3 Dendrogram of *E. faecalis* based on PFGE

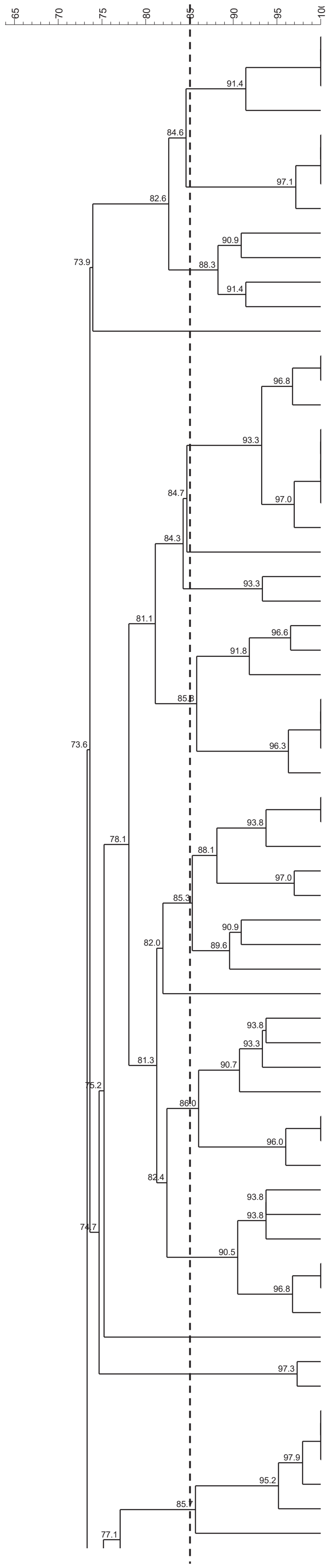

Strain code	Sample Matrix	Sample Subject	Farm	Antibiotic Resistance Profiles	Biofilm Formation	
P.En012	Oral	Pig	6	E15, TET30, CIP5, C30, CN10, CN120	F	
P.En042	Rectal	Pig	6	E15, TET30, CIP5, C30, LZD30, CN10, CN120	N	C1
P.En046	Rectal	Pig	6	E15, TET30, CIP5, ENR5, C30, CN10, CN120	F	
Enfs63	Oral	Pig	2	E15, TET30, CIP5, C30, CN10, CN120	F	
Enfs51	Fecal	Pig	2	E15, TET30, CIP5, C30, LZD30, CN10, CN120	F	
Enfs52	Fecal	Pig	2	E15, TET30, CIP5, C30, CN10, CN120	F	C2
Enfs58	Fecal	Pig	2	E15, TET30, CIP5, C30, CN10, CN120	F	
Enfs116	Rectal	Pig	2	E15, TET30, CIP5, C30, LZD30, CN10, CN120	F	
Enfs130	Rectal	Pig	3	E15, TET30, CIP5, C30, LZD30, CN10, CN120	F	
Enfs135	Rectal	Pig	3	E15, TET30, CIP5, C30, CN10, CN120	F	C3
Enfs136	Rectal	Pig	3	E15, TET30, CIP5, C30, CN10, CN120	F	
Enfs137	Rectal	Pig	3	E15, TET30, CIP5, C30, CN10, CN120	N	
Enfs87	Fecal	Pig	3	E15, TET30, CIP5, C30, LZD30, CN10	F	
Enfs122	Rectal	Pig	2	E15, TET30, CIP5, C30, LZD30, CN10, CN120	F	
Enfs124	Rectal	Pig	2	E15, TET30, CIP5, C30, LZD30, CN10, CN120	F	
Enfs103	Floor	Environme.	2	E15, TET30, CIP5, C30, LZD30, CN10, CN120	F	
Enfs119	Rectal	Pig	2	E15, TET30, CIP5, C30, LZD30, CN10	F	C4
Enfs123	Rectal	Pig	2	E15, TET30, CIP5, C30, LZD30, CN10, CN120	F	
Enfs153	Nasal	Human	4	P10, E15, TET30, CIP5, C30, LZD30, CN10, CN120	F	
Enfs57	Fecal	Pig	2	E15, TET30, CIP5, C30, LZD30, CN10	F	
Enfs76	Oral	Pig	2	E15, TET30, CIP5, C30, LZD30, CN10, CN120	F	
P.En057	Oral	Pig	6	E15, TET30, CIP5, ENR5, C30, CN10, CN120	F	
Enfs125	Rectal	Pig	2	E15, TET30, CIP5, C30, LZD30, CN10	F	C5
Enfs60	Fecal	Pig	2	E15, TET30, CIP5, C30, CN10	F	
Enfs120	Rectal	Pig	2	E15, TET30, CIP5, C30, LZD30, CN10, CN120	F	
Enfs55	Fecal	Pig	2	E15, TET30, CIP5, C30, LZD30, CN10, CN120	F	
Enfs98	Water	Environme.	3	E15, TET30, CIP5, C30, CN10	F	
P.En054	Rectal	Pig	6	E15, TET30, CIP5, C30, CN10, CN120	F	C6
P.En055	Rectal	Pig	6	E15, TET30, CIP5, ENR5, C30, CN10, CN120	F	
P.En074	Rectal	Pig	6	E15, TET30, CIP5, C30, CN10, CN120	F	
P.En004	Rectal	Pig	6	E15, TET30, CIP5, C30, CN10, CN120	F	
P.En092	Nasal	Human	6	TEC30, E15, TET30, CIP5, ENR5, C30, LZD30, CN10, CN120	F	
P.En093	Nasal	Human	6	E15, CIP5, ENR5, C30, LZD30, CN10, CN120	F	
P.En145	Oral	Pig	7	P10, E15, TET30, CIP5, C30, CN10, CN120	F	
Enfs100	Rectal	Pig	5	E15, TET30, CIP5, C30, LZD30, CN10, CN120	F	C7
Enfs150	Rectal	Pig	5	E15, TET30, CIP5, C30, LZD30, CN10	N	
Enfs88	Fecal	Pig	3	E15, TET30, CIP5, C30, LZD30, CN10,	F	
P.En006	Oral	Pig	6	E15, TET30, CIP5, C30, CN10, CN120	F	
P.En075	Rectal	Pig	6	E15, TET30, CIP5, C30, CN10, CN120	F	
Enfs149	Rectal	Pig	5	E15, TET30, CIP5, C30	N	
Enfs37	Rectal	Pig	1	E15, TET30, CIP5, C30, LZD30	N	
Enfs38	Rectal	Pig	1	E15, TET30, CIP5, C30, LZD30, CN10	N	
Enfs134	Rectal	Pig	3	E15, TET30, CIP5, C30, CN10	N	
Enfs53	Fecal	Pig	2	E15, TET30, CIP5, C30, LZD30	N	C8
Enfs85	Fecal	Pig	1	E15, TET30, CIP5, C30, LZD30, CN10, CN120	F	
Enfs86	Fecal	Pig	1	E15, TET30, CIP5, C30, LZD30, CN10	F	
Enfs35	Rectal	Pig	1	E15, TET30, CIP5, C30, CN10, CN120	F	
Enfs114	Nasal	Human	4	E15, TET30, CIP5, C30, LZD30, CN10	N	
Enfs148	Rectal	Pig	5	E15, TET30, CIP5, C30, LZD30, CN10, CN120	N	
Enfs97	Nasal	Human	4	E15, TET30, C30, LZD30, CN10, CN120	F	C9
Enfs66	Oral	Pig	4	E15, TET30, CIP5, C30, CN10	F	
P.En098	Nasal	Human	6	E15, TET30, CIP5, ENR5, C30, LZD30, CN10, CN120	N	
P.En112	Nasal	Human	6	AMP10, E15, TET30, CIP5, ENR5, C30, LZD30, CN10	N	
P.En079	Oral	Pig	6	E15, TET30, CIP5, C30, LZD30, CN10	N	
P.En020	Rectal	Pig	6	E15, TET30, CIP5, C30, CN10, CN120	F	C10
P.En201	Oral	Pig	7	E15, TET30, CIP5, C30, LZD30, CN10	F	
Enfs131	Rectal	Pig	3	E15, TET30, CIP5, C30, LZD30, CN10	N	
Enfs90	Fecal	Pig	3	E15, TET30, CIP5, C30, LZD30, CN10	N	
Enfs91	Oral	Pig	1	E15, TET30, CIP5, C30, LZD30, CN10	N	C11
Enfs89	Fecal	Pig	3	P10, E15, TET30, CIP5, C30, LZD30, CN10	N	
Enfs64	Oral	Pig	2	E15, TET30, CIP5, C30, LZD30, CN10	F	
Enfs65	Oral	pig	2	E15, TET30, CIP5, C30, LZD30, CN10	N	

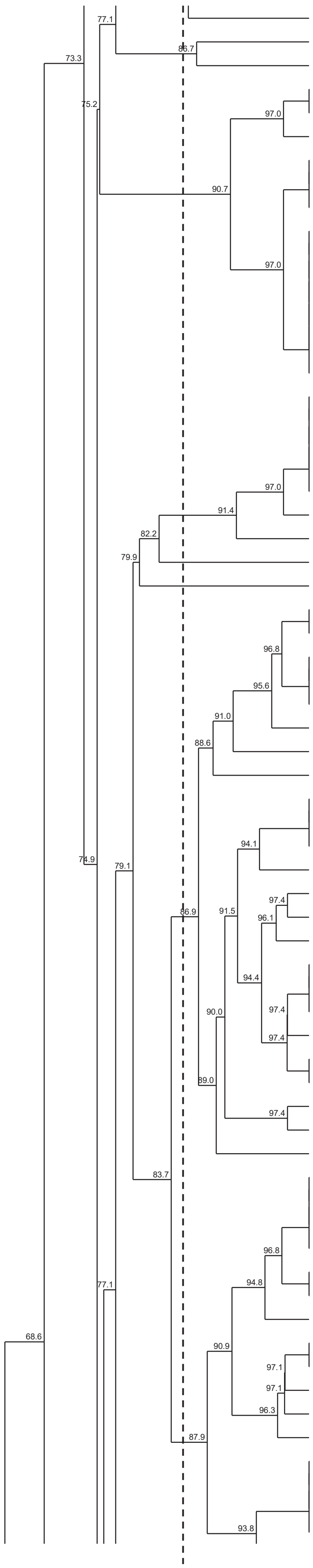

Enfs65	Oral	pig	2	E15, TET30, CIP5, C30, LZD30, CN10	N	
Enfs102	Urine	Human	4	E15, TET30, C30, LZD30, CN10	F	C12
Enfs59	Fecal	Pig	2	E15, TET30, CIP5, C30, CN10, CN120	F	
P.En180	Oral	Pig	7	E15, TET30, CIP5, C30, LZD30, CN10, CN120	F	
P.En181	Oral	Pig	7	E15, TET30, CIP5, C30, LZD30, CN10, CN120	N	
P.En182	Rectal	Pig	7	E15, TET30, CIP5, C30, LZD30, CN10, CN120	N	
P.En202	Rectal	Pig	7	E15, TET30, CIP5, C30, LZD30, CN10, CN120	F	
P.En215	Urine	Human	7	E15, TET30, CIP5, ENR5, C30, LZD30, CN10	F	
P.En218	Fecal	Human	7	E15, TET30, CIP5, ENR5, C30, LZD30, CN10, CN120	N	
P.En120	Nasal	Human	7	E15, TET30, CIP5, ENR5, C30, LZD30, CN10, CN120	F	C13
P.En168	Oral	Pig	7	E15, TET30, CIP5, C30, LZD30, CN10, CN120	F	
P.En170	Rectal	Pig	7	E15, TET30, CIP5, ENR5, C30, LZD30, CN10, CN120	N	
P.En172	Rectal	Pig	7	E15, TET30, CIP5, ENR5, C30, LZD30, CN10, CN120	F	
P.En173	Rectal	Pig	7	E15, TET30, CIP5, C30, LZD30, CN10, CN120	F	
P.En186	Oral	Pig	7	E15, TET30, CIP5, C30, LZD30, CN10, CN120	N	
P.En197	Oral	Pig	7	E15, TET30, CIP5, C30, LZD30, CN10, CN120	N	
Enfs105	Urine	Human	4	E15, TET30, CIP5, C30, LZD30, CN10, CN120	N	
Enfs106	Urine	Human	4	E15, TET30, CIP5, C30, LZD30, CN10, CN120	F	
Enfs107	Urine	Human	4	E15, TET30, CIP5, C30, LZD30, CN10, CN120	N	
Enfs112	Urine	Human	4	E15, TET30, CIP5, C30, LZD30, CN10, CN120	F	C14
ENfs95	Urine	Human	4	E15, TET30, CIP5, C30, LZD30, CN10, CN120	F	
Enfs152	Urine	Human	4	E15, TET30, CIP5, C30, LZD30, CN10, CN120	N	
Enfs61	Oral	Pig	4	TEC30, E15, TET30, CIP5, C30, LZD30, CN10, CN120	F	
Enfs62	Oral	Pig	5	E15, TET30, CIP5, C30, LZD30, CN10	F	
P.En086	Oral	Pig	6	E15, TET30, CIP5, ENR5, C30, LZD30, CN10, CN120	F	
P.En062	Rectal	Pig	6	E15, TET30, CIP5, ENR5, C30, CN10, CN120	F	
P.En178	Rectal	Pig	7	E15, TET30, CIP5, C30, LZD30, CN10, CN120	F	
Enfs69	Oral	Pig	1	E15, TET30, CIP5, C30, CN10, CN120	F	
Enfs70	Oral	Pig	1	E15, TET30, CIP5, C30, LZD30, CN10, CN120	F	
P.En083	Oral	Pig	6	E15, TET30, CIP5, C30, LZD30, CN10, CN120	F	
Enfs67	Oral	Pig	1	E15, TET30, CIP5, C30, CN10, CN120	F	
Enfs94	Fecal	Human	1	E15, TET30, CIP5, LZD30, CN10	F	
P.En174	Oral	Pig	7	E15, TET30, CIP5, C30, LZD30, CN10, CN120	F	
Enfs73	Oral	Pig	1	E15, TET30, C30, LZD30, CN10, CN120	N	
Enfs74	Oral	Pig	1	E15, TET30, C30, LZD30, CN10, CN120	F	
Enfs75	Oral	Pig	1	E15, TET30, C30, LZD30, CN10, CN120	F	
P.En018	Oral	Pig	6	E15, TET30, CIP5, ENR5, C30, CN10, CN120	F	C15
Enfs96	Nasal	Human	4	E15, TET30, C30, LZD30, CN10, CN120	F	
P.En287	Urine	Human	6	E15, TET30, CIP5, ENR5, C30, LZD30, CN10, CN120	F	
P.En284	Urine	Human	6	E15, TET30, CIP5, ENR5, C30, LZD30, CN10, CN120	F	
P.En094	Nasal	Human	6	E15, TET30, CIP5, ENR5, C30, LZD30, CN10, CN120	F	
P.En143	Rectal	Pig	7	E15, TET30, CIP5, ENR5, C30, LZD30, CN10, CN120	F	
P.En142	Rectal	Pig	7	E15, TET30, CIP5, C30, LZD30, CN10, CN120	F	
Enfs104	Nasal	Human	3	E15, TET30, CIP5, C30, LZD30, CN10, CN120	F	
Enfs101	Urine	Human	3	E15, TET30, CIP5, C30, LZD30, CN10, CN120	F	
ENfs99	Fence	Environme.	1	E15, TET30, C30, LZD30, CN10, CN120	F	
P.En275	Urine	Human	6	E15, TET30, CIP5, ENR5, C30, LZD30, CN10, CN120	F	
P.En283	Urine	Human	6	E15, TET30, CIP5, ENR5, C30, LZD30, CN10, CN120	F	
Enfs111	Urine	Human	5	E15, TET30, CIP5, C30, LZD30, CN10, CN120	N	
P.En195	Rectal	Pig	7	E15, TET30, CIP5, C30, LZD30, CN10, CN120	N	
P.En224	Fecal	Human	7	E15, TET30, CIP5, ENR5, C30, CN10	N	
P.En226	Fecal	Human	7	E15, TET30, CIP5, ENR5, CN10	N	
P.En249	Fecal	Pig	7	E15, TET30, CIP5, ENR5, C30, LZD30, CN10, CN120	F	
P.En084	Rectal	Pig	6	E15, TET30, CIP5, C30, LZD30, CN10, CN120	N	
P.En110	Nasal	Human	6	E15, TET30, CIP5, ENR5, C30, LZD30, CN10, CN120	N	
P.En223	Fecal	Human	7	E15, TET30, CIP5, ENR5, C30, CN10	F	
Enfs03	Fecal	Pig	5	E15, TET30, CIP5, C30, LZD30, CN10	F	C16
Enfs04	Fecal	Pig	5	E15, TET30, CIP5, C30, LZD30, CN10	F	
Enfs113	Rectal	Pig	5	E15, TET30, CIP5, C30, LZD30, CN10	N	
Enfs12	Fecal	Pig	4	E15, TET30, CIP5, C30, LZD30, CN10	F	
Enfs10	Fecal	Pig	4	E15, TET30, CIP5, C30, LZD30, CN10	F	
P.En187	Oral	Pig	7	E15, TET30, CIP5, C30, LZD30, CN10, CN120	N	
P.En214	Urine	Human	7	E15, TET30, CIP5, ENR5, C30, LZD30, CN10	F	
P.En220	Fecal	Human	7	E15, TET30, CIP5, ENR5, C30, LZD30, CN10, CN120	N	
P.En229	Fecal	Pig	6	E15, TET30, CIP5, C30, LZD30, CN10, CN120	F	

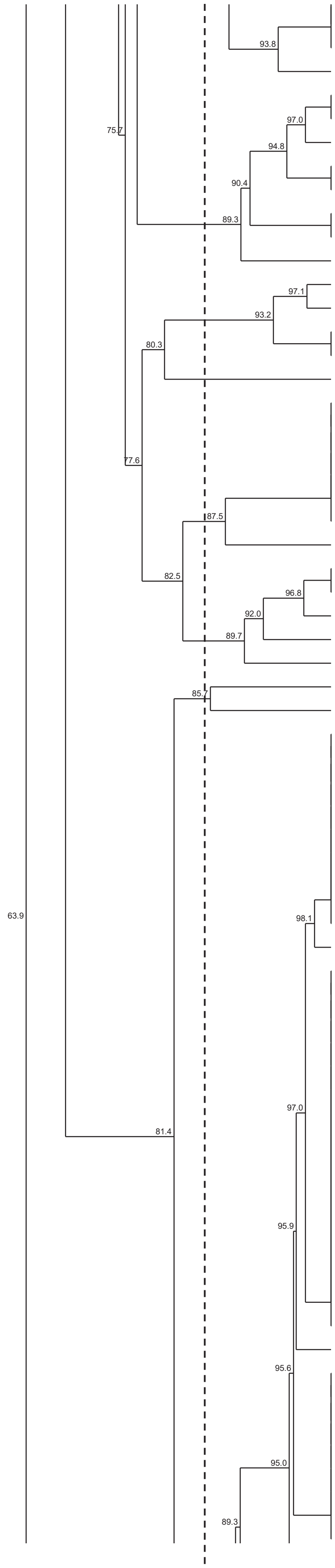

P.En220	Fecal	Human	7	E15, TET30, CIP5, ENR5, C30, LZD30, CN10, CN120	N	C16
P.En229	Fecal	Pig	6	E15, TET30, CIP5, C30, LZD30, CN10, CN120	F	
P.En217	Urine	Human	7	E15, TET30, CIP5, ENR5, C30, LZD30, CN10	F	
P.En024	Oral	Pig	6	E15, TET30, CIP5, ENR5, C30, LZD30, CN10, CN120	N	C17
P.En028	Oral	Pig	6	E15, TET30, CIP5, ENR5, C30, LZD30, CN10, CN120	N	
Enfs128	Rectal	Pig	2	E15, TET30, CIP5, C30, LZD30, CN10, CN120	F	
P.En022	Oral	Pig	6	E15, TET30, CIP5, C30, LZD30, CN10, CN120	F	
P.En152	Oral	Pig	7	E15, TET30, CIP5, C30, LZD30, CN10, CN120	F	
P.En146	Rectal	Pig	7	E15, TET30, CIP5, C30, LZD30, CN10, CN120	F	
P.En147	Rectal	Pig	7	E15, TET30, CIP5, C30, LZD30, CN10, CN120	N	
Enfs138	Rectal	Pig	4	E15, TET30, C30, LZD30, CN10	F	
Enfs02	Fecal	Pig	5	E15, TET30, CIP5, C30, CN10, CN120	F	C18
Enfs08	Fecal	Pig	4	E15, TET30, CIP5, C30, CN10, CN120	F	
Enfs01	Fecal	Pig	5	E15, TET30, CIP5, C30, CN10, CN120	F	
Enfs07	Fecal	Pig	4	E15, TET30, CIP5, C30, CN10, CN120	F	
Enfs56	Fecal	Pig	2	E15, TET30, CIP5, C30, CN10	N	
Enfs05	Fecal	Pig	5	E15, TET30, C30, CN10	F	C19
Enfs13	Fecal	Pig	4	E15, TET30, C30, CN10	F	
Enfs14	Fecal	Pig	4	P10, E15, TET30, CIP5, C30, CN10	F	
Enfs15	Fecal	Pig	4	E15, TET30, C30, CN10	N	
Enfs16	Fecal	Pig	4	E15, TET30, C30, CN10	F	
Enfs17	Rectal	Pig	4	E15, TET30, C30, CN10	F	
Enfs45	Rectal	Pig	1	E15, TET30, CIP5, C30, LZD30, CN10, CN120	N	
Enfs43	Rectal	Pig	1	E15, TET30, CIP5, C30, CN10	N	C20
Enfs46	Rectal	Pig	1	E15, TET30, CIP5, C30, LZD30, CN10	N	
Enfs71	Oral	Pig	1	P10, E15, TET30, CIP5, C30, CN10	N	
Enfs72	Oral	Pig	1	E15, TET30, CIP5, C30, CN10	N	
Enfs92	Oral	Pig	1	E15, TET30, CIP5, C30, CN10	N	
Enfs117	Rectal	Pig	2	E15, TET30, CIP5, C30, LZD30, CN10	N	C21
Enfs30	Rectal	Pig	1	E15, TET30, CIP5, C30, LZD30, CN10, CN120	N	
Enfs126	Rectal	Pig	2	E15, TET30, CIP5, C30, LZD30, CN10, CN120	F	C22
Enfs36	Rectal	Pig	1	E15, TET30, CIP5, C30, LZD30, CN10, CN120	F	
Enfs40	Rectal	Pig	1	E15, TET30, CIP5, C30, LZD30, CN10, CN120	F	
Enfs41	Rectal	Pig	1	E15, TET30, CIP5, C30, CN10, CN120	F	
Enfs47	Rectal	Pig	1	E15, TET30, CIP5, C30, CN10, CN120	N	
Enfs48	Rectal	Pig	1	E15, TET30, CIP5, C30, LZD30, CN10, CN120	F	
Enfs49	Rectal	Pig	1	E15, TET30, CIP5, C30, LZD30, CN10, CN120	F	
Enfs82	Fecal	Pig	2	E15, TET30, CIP5, C30, CN10, CN120	N	
Enfs83	Fecal	Pig	2	E15, TET30, CIP5, C30, LZD30, CN10, CN120	N	
Enfs26	Rectal	Pig	1	E15, TET30, CIP5, C30, CN10, CN120	N	
Enfs129	Rectal	Pig	2	E15, TET30, CIP5, C30, LZD30, CN10, CN120	F	
Enfs18	Rectal	Pig	4	E15, TET30, C30, CN10	N	
Enfs23	Fecal	Pig	1	E15, TET30, CIP5, C30, LZD30, CN10, CN120	N	
Enfs24	Fecal	Pig	1	E15, TET30, CIP5, C30, CN10, CN120	F	
Enfs25	Rectal	Pig	1	E15, TET30, CIP5, C30, CN10, CN120	N	
Enfs27	Rectal	Pig	1	E15, TET30, CIP5, C30, LZD30, CN10, CN120	N	
Enfs29	Rectal	Pig	1	E15, TET30, CIP5, C30, LZD30, CN10, CN120	F	
Enfs32	Rectal	Pig	1	E15, TET30, CIP5, C30, LZD30, CN10, CN120	F	
Enfs33	Rectal	Pig	1	E15, TET30, CIP5, C30, CN10	F	
Enfs34	Rectal	Pig	1	E15, TET30, CIP5, C30, CN10, CN120	N	
Enfs39	Rectal	Pig	1	E15, TET30, CIP5, C30, LZD30, CN10, CN120	N	
Enfs50	Rectal	Pig	1	E15, TET30, CIP5, C30, LZD30, CN10	N	
Enfs78	Fecal	Pig	2	E15, TET30, CIP5, C30, LZD30, CN10, CN120	F	
Enfs81	Fecal	Pig	2	E15, TET30, CIP5, C30, LZD30, CN10, CN120	N	
Enfs84	Fecal	Pig	2	E15, TET30, CIP5, C30, CN10, CN120	N	
P.En164	Rectal	Pig	7	E15, TET30, CIP5, C30, LZD30, CN10	F	
Enfs80	Fecal	Pig	2	E15, TET30, CIP5, C30, CN10, CN120	N	
Enfs22	Fecal	Pig	1	E15, TET30, CIP5, C30, LZD30, CN10, CN120	N	
P.En070	Rectal	Pig	6	E15, TET30, CIP5, C30, CN10, CN120	F	
P.En071	Rectal	Pig	6	E15, TET30, CIP5, C30, LZD30, CN10, CN120	F	
P.En090	Rectal	Pig	6	E15, TET30, CIP5, ENR5, C30, CN10, CN120	F	
P.En148	Oral	Pig	7	E15, TET30, CIP5, C30, LZD30, CN10, CN120	F	
P.En150	Rectal	Pig	7	E15, TET30, CIP5, ENR5, C30, LZD30, CN10, CN120	F	
P.En154	Rectal	Pig	7	E15, TET30, CIP5, C30, LZD30, CN10, CN120	F	
P.En155	Rectal	Pig	7	E15, TET30, CIP5, ENR5, C30, LZD30, CN10, CN120	F	

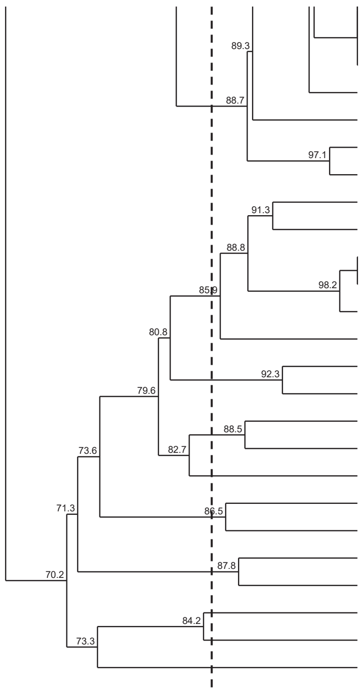

P.En150	Rectal	Pig	7	E15, TET30, CIP5, ENR5, C30, LZD30, CN10, CN120	F	C22
P.En154	Rectal	Pig	7	E15, TET30, CIP5, C30, LZD30, CN10, CN120	F	
P.En155	Rectal	Pig	7	E15, TET30, CIP5, ENR5, C30, LZD30, CN10, CN120	F	
Enfs31	Rectal	Pig	1	E15, TET30, CIP5, C30, LZD30, CN10, CN120	N	
P.En008	Rectal	Pig	6	E15, TET30, CIP5, C30, CN10, CN120	F	
P.En003	Rectal	Pig	6	E15, TET30, CIP5, C30, LZD30, CN10, CN120	F	
P.En043	Rectal	Pig	6	E15, TET30, CIP5, C30, LZD30, CN10, CN120	N	
P.En203	Rectal	Pig	7	E15, TET30, CIP5, C30, LZD30, CN10, CN120	F	C23
P.En242	Fecal	Pig	6	AMP10, P10, E15, TET30, CIP5, ENR5, C30, LZD30, CN10, CN120	N	
P.En207	Oral	Pig	7	E15, TET30, C30	F	
P.En208	Oral	Pig	7	E15, TET30	N	
P.En204	Oral	Pig	7	E15, TET30, CIP5, C30, LZD30, CN10, CN120	F	
P.En052	Oral	Pig	6	AMP10, P10, E15, TET30, CIP5, C30, CN10, CN120	N	
P.En246	Fecal	Pig	7	P10, E15, TET30, CIP5, ENR5, C30	F	C24
P.En251	Fecal	Pig	7	AMP10, P10, E15, TET30, CIP5, C30, LZD30, CN10, CN120	N	
P.En091	Rectal	Pig	6	TEC30, E15, TET30, CIP5, ENR5, LZD30, CN10, CN120	N	C25
P.En243	Fecal	Pig	7	AMP10, E15, TET30, CIP5, ENR5, C30, LZD30	N	
P.En259	Fecal	Human	6	E15, ENR5, C30	N	
P.En191	Oral	Pig	7	E15, TET30, CIP5, C30, LZD30, CN10, CN120	F	C26
P.En250	Fecal	Pig	7	E15, TET30, CIP5, ENR5, C30, LZD30, CN10, CN120	F	
P.En192	Rectal	Pig	7	E15, TET30, CIP5, ENR5, C30, LZD30, CN10, CN120	F	C27
P.En244	Fecal	Pig	7	E15, TET30, CIP5, ENR5, C30, CN10, CN120	N	
P.En011	Oral	Pig	6	E15, TET30, CIP5, CN10, CN120	N	
P.En029	Oral	Pig	6	E15, TET30, CIP5, CN10, CN120	N	
P.En225	Fecal	Human	7	TET30, CIP5, ENR5, C30, LZD30, CN10	N	
